# Treatment of chronic axial back pain with 60‐day percutaneous medial branch PNS: Primary end point results from a prospective, multicenter study

**DOI:** 10.1111/papr.13055

**Published:** 2021-07-27

**Authors:** Christopher A. Gilmore, Mehul J. Desai, Thomas J. Hopkins, Sean Li, Michael J. DePalma, Timothy R. Deer, Warren Grace, Abram H. Burgher, Puneet K. Sayal, Kasra Amirdelfan, Steven P. Cohen, Meredith J. McGee, Joseph W. Boggs

**Affiliations:** ^1^ Center for Clinical Research Winston Salem North Carolina USA; ^2^ International Spine, Pain, and Performance Center Washington DC USA; ^3^ Duke University Durham North Carolina USA; ^4^ Premier Pain Centers Shrewsbury New Jersey USA; ^5^ Virginia iSpine Physicians Richmond Virginia USA; ^6^ The Spine and Nerve Centers of the Virginias Charleston West Virginia USA; ^7^ The Pain Center Phoenix Arizona USA; ^8^ Integrated Pain Management, Inc Walnut Creek California USA; ^9^ Johns Hopkins School of Medicine Baltimore Maryland USA; ^10^ SPR Therapeutics, Inc Cleveland Ohio USA

**Keywords:** axial low back pain, chronic back pain, medial branch stimulation, multifidus activation, neuromodulation, non‐opioid, percutaneous peripheral nerve stimulation (PNS), peripheral nerve stimulation (PNS)

## Abstract

**Background:**

The objective of this prospective, multicenter study is to characterize responses to percutaneous medial branch peripheral nerve stimulation (PNS) to determine if results from earlier, smaller single‐center studies and reports were generalizable when performed at a larger number and wider variety of centers in patients recalcitrant to nonsurgical treatments.

**Materials & Methods:**

Participants with chronic axial low back pain (LBP) were implanted with percutaneous PNS leads targeting the lumbar medial branch nerves for up to 60 days, after which the leads were removed. Participants were followed long‐term for 12 months after the 2‐month PNS treatment. Data collection is complete for visits through end of treatment with PNS (primary end point) and 6 months after lead removal (8 months after start of treatment), with some participant follow‐up visits thereafter in progress.

**Results:**

Clinically and statistically significant reductions in pain intensity, disability, and pain interference were reported by a majority of participants. Seventy‐three percent of participants were successes for the primary end point, reporting clinically significant (≥30%) reductions in back pain intensity after the 2‐month percutaneous PNS treatment (*n* = 54/74). Whereas prospective follow‐up is ongoing, among those who had already completed the long‐term follow‐up visits (*n* = 51), reductions in pain intensity, disability, and pain interference were sustained in a majority of participants through 14 months after the start of treatment.

**Conclusion:**

Given the minimally invasive, nondestructive nature of percutaneous PNS and the significant benefits experienced by participants who were recalcitrant to nonsurgical treatments, percutaneous PNS may provide a promising first‐line neurostimulation treatment option for patients with chronic axial back pain.

**Key Points**

- Chronic low back pain is one of the most prevalent and challenging musculoskeletal conditions and is the leading cause of disability in adults. The present prospective, multicenter study was conducted to characterize responses to percutaneous medial branch peripheral nerve stimulation (PNS) among participants with chronic axial low back pain.
- Clinically and statistically significant reductions in pain, disability, and pain interference were reported by a majority of participants with percutaneous PNS, along with reductions in opioid consumption and statistically significant improvements in health‐related Quality of Life.
- Given the minimally invasive, non‐destructive nature of percutaneous PNS and the significant benefits, percutaneous PNS may provide a promising first‐line neurostimulation treatment for patients with chronic axial LBP.

## INTRODUCTION

Neurostimulation offers a pain management solution for many chronic pain conditions, including chronic axial low back pain (LBP), that are difficult to treat with traditional approaches, such as medication management, radiofrequency ablation, and/or surgery.^1^, ^2^ In patients with chronic LBP, neurostimulation has been shown to provide clinically significant reductions in pain, opioid use, and disability.^3^, ^4^, ^5^, ^6^, ^7^, ^8^, ^9^, ^10^, ^11^, ^12^ However, implanted neurostimulation systems are typically used late in the treatment continuum as a last‐resort therapy,^3^, ^12^, ^13^, ^14^ and their limited use may be attributed to the risks and patient aversion to permanent implantation of such systems.^15^, ^16^, ^17^, ^18^, ^19^, ^20^, ^21^ Alternately, a temporary, minimally invasive neurostimulation treatment applied to peripheral nerves over a 60‐day treatment period (i.e., percutaneous peripheral nerve stimulation [PNS]) is a promising non‐opioid, nondestructive, and nonsurgical treatment for chronic axial LBP. Percutaneous PNS offers the potential to avoid the patient aversion, cost, invasiveness, and challenges associated with permanently implanted neurostimulation systems, and addresses the shortcomings associated with traditional approaches (e.g., avoiding dependence on opioids, providing a nondestructive and reversible treatment, and avoiding surgery).

Percutaneous PNS was designed as a temporary (60‐day duration) treatment that is implanted without surgery via a small gauge percutaneous introducer to deliver stimulation via remote‐targeting of peripheral nerves, avoiding the need for invasive procedures to place stimulation electrodes in close proximity to nerve tissue and permanent implantation of system components, as is typical with other neurostimulation (e.g., both conventional spinal cord stimulation [SCS] or PNS) systems. A review of more than 16 publications of percutaneous PNS, representing 12 clinical studies (3 randomized controlled trials, 6 prospective case series, and 3 case reports) found clinically significant reductions across a range of patient‐centric outcomes, including pain, disability, and opioid consumption among patients with a variety of chronic pain conditions.^22^, ^23^, ^24^, ^25^, ^26^, ^27^, ^28^, ^29^, ^30^, ^31^, ^32^, ^33^, ^34^, ^35^, ^36^, ^37^ Reductions in pain and/or pain interference were sustained at both 3 months (77%, *n* = 62/81) and 1 year (76%, *n* = 35/46) following percutaneous PNS across the studies examined, demonstrating sustained, clinically meaningful improvements following the temporary 60‐day PNS treatment.

However, prior publications and presentations of results with percutaneous PNS for the treatment of chronic axial back pain have been limited to individual case reports or small, single center case series studies.^34^, ^36^, ^38^, ^39^, ^40^ Given the prevalence and societal burden of chronic axial back pain,^1^, ^41^, ^42^ there is a need for larger, well‐designed clinical trials to determine best practices for application of PNS, and a recent systematic review specifically called for additional prospective studies.^43^ The goal of this clinical study was to investigate the potential for percutaneous PNS to relieve pain and improve functional outcomes among patients with axial LBP who had failed multiple nonsurgical treatments to determine if the results from earlier, smaller, single‐center studies were generalizable when performed by physicians with varied specialties and techniques, across a wide variety of centers (e.g., large academic centers, research institutions, and private practice clinics) in settings with rural and urban populations. Although preliminary data in a small number of patients who had a return of pain after radiofrequency ablation (RFA) have been recently reported,^44^ the present paper is the first to report results of the largest multicenter clinical study to date of percutaneous PNS among the full cohort of participants with chronic axial LBP recalcitrant to multiple nonsurgical treatments through the prospectively defined primary end point.

## MATERIALS AND METHODS

Patients with chronic axial LBP were screened for participation in this institutional review board approved (IRB; Quorum Review IRB) prospective, multicenter case series study (registered on ClinicalTrials.gov, NCT03179202) conducted across a variety of clinical care settings. Written informed consent was obtained from each participant. The inclusion criteria required participants to have chronic axial LBP (i.e., report pain with a score ≥4 on a 0–10 scale that was confined to the lumbar region and had lasted at least 12 weeks), failure of at least 2 different categories of LBP treatments (e.g., medications, physical therapy, and injections), and at least 4 weeks of stable analgesic medication usage. The exclusion criteria included radicular leg pain, prior lumbar surgery,^1^ lumbar anesthetic injections within 3 months of baseline (apart from diagnostic medial branch blocks), lumbar RFA within 6 months of baseline, lumbar scoliosis, pending secondary gain issues, allergy to adhesives, body mass index (BMI) greater than or equal to 40, a score greater than 20 on the Beck Depression Inventory (BDI‐II) indicating moderate depression, and conditions contraindicated by the PNS system’s instructions for use (IFU). A physical examination was conducted at baseline to confirm eligibility and collect back pain‐related history (e.g., review of back pain treatment history, prior imaging, or diagnostic testing). Aside from the inclusion and exclusion criteria, there were no specific requirements for pain of a specific etiology, and participants with various etiologies of axial pain were potentially eligible for inclusion (e.g., spondylosis, degenerative disc disease, facetogenic pain, nonspecific back pain, etc.). To confirm the back pain score reported at the baseline visit, participants completed a 7‐day written diary of daily average back pain intensity (Brief Pain Inventory, Question #5 [BPI‐5]), and must have reported a mean score greater than or equal to 4 to qualify for PNS lead implantation.

### PNS lead implant

**FIGURE 1:**
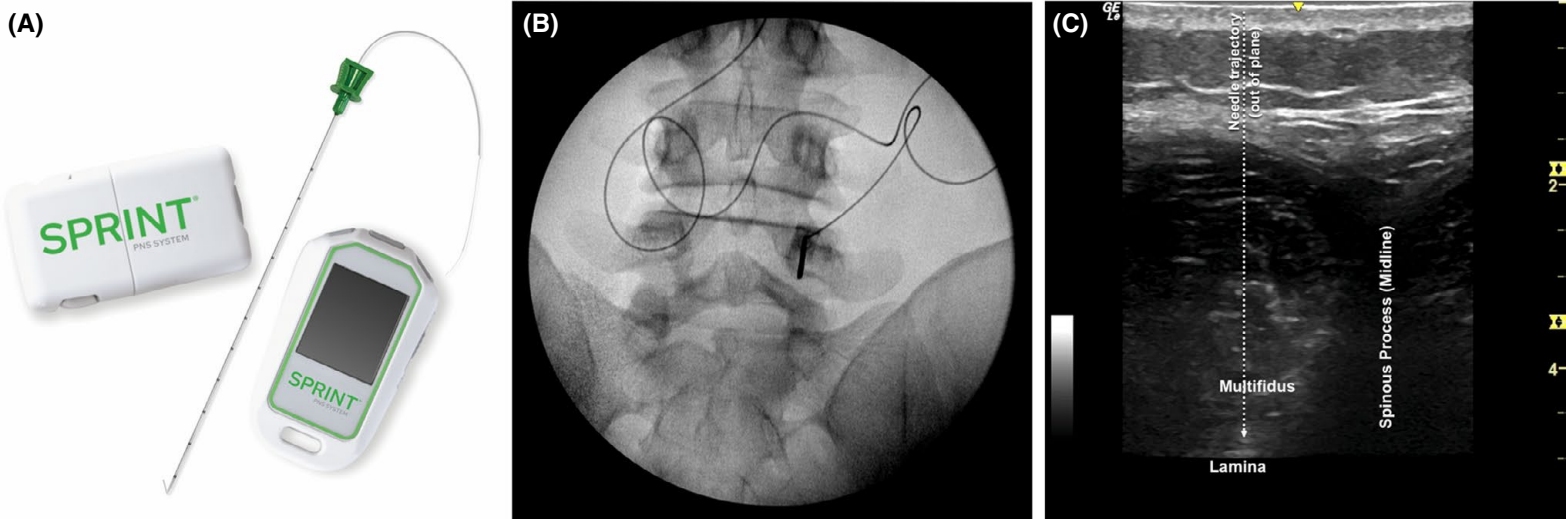
Implantation of percutaneous PNS targeting the medial branch nerves via ultrasound and/or fluoroscopic guidance for chronic axial back pain. Participants received percutaneous PNS (SPRINT PNS System, SPR Therapeutics, Inc) (a) targeting the medial branches of the dorsal rami at the spinal level in the center of the region of axial back pain. All leads were placed using ultrasound and/or fluoroscopic image guidance. (b) Shows stimulation test probe insertion targeting the medial branches of the dorsal rami, medial, and inferior to the facet joint, with an anteroposterior (AP) fluoroscopic view. (c) Shows an ultrasound image of the lumbar paraspinal anatomy (as viewed with a transverse probe orientation), illustrating example out‐of‐plane PNS lead implantation to target the medial branch of the dorsal ramus over lamina. Ultrasound guidance enabled visualization of multifidus muscle responses, confirming selective activation of the medial branch nerves with PNS in all participants. PNS, peripheral nerve stimulation

**FIGURE 2:**
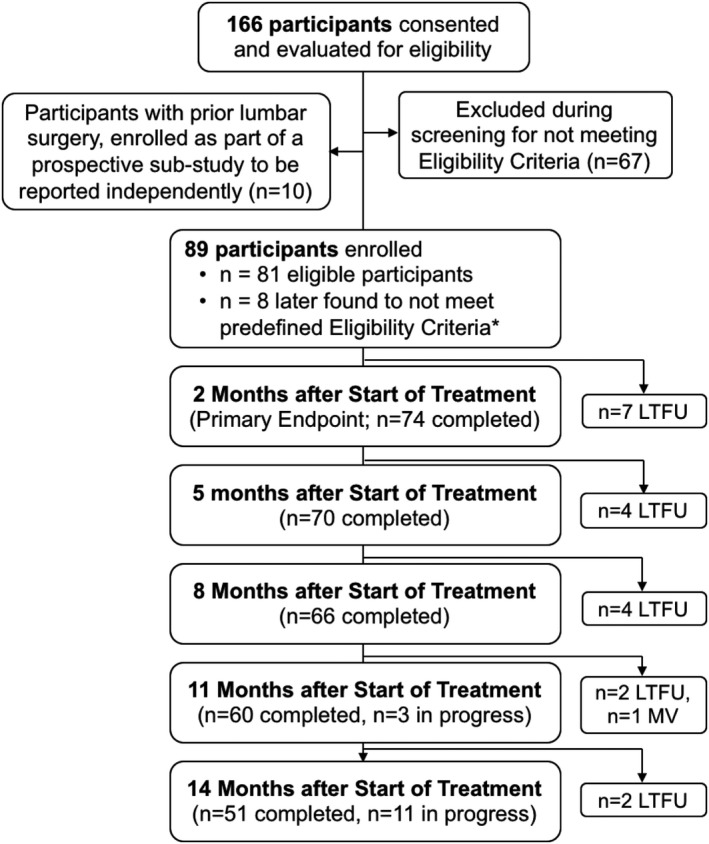
Subject participation flow diagram. Participants with chronic axial low back pain were consented, evaluated for eligibility, enrolled, and underwent implantation of percutaneous PNS. Ten participants were enrolled under this study’s IRB approval as part of a prospectively designed substudy with revised eligibility criteria, designed to be reported independently from the primary end point analysis of participants without a history of prior lumbar surgery, and are not analyzed here. Eight participants were not included in the primary end point analysis after monitoring identified they did not meet the prospectively defined eligibility criteria at baseline (e.g., radicular leg pain, new medications at baseline, and scoliosis). * Of the 81 eligible participants receiving PNS leads, 74 completed the PNS treatment and primary end point (2 months after start of treatment) and 7 participants were lost to follow‐up. Prospective follow‐up is ongoing and will continue until all participants reach 14 months (12 months after end of treatment with PNS). *A post hoc sensitivity analysis confirmed that not including participants who did not meet the predefined eligibility criteria at baseline did not have a meaningful impact on interpretation of the primary end point results (see results: Primary End Point and Reductions in Pain Intensity section). IRB, institutional review board; LTFU, lost to follow‐up; MV, missed visit; PNS, peripheral nerve stimulation

After mapping the location and distribution of each participant’s axial back pain, percutaneous open‐coil PNS leads (MicroLead; SPR Therapeutics, Inc; Figure 1a) were implanted bilaterally under ultrasound and/or fluoroscopic guidance to target the medial branch nerves at the vertebral level in the center of the region of pain. At the level of interest, the PNS lead was typically introduced 1–2 cm lateral from midline at an angle of approximately 90 degrees to the skin to a depth of 4–6 cm, depending on body habitus, to target the medial branch nerves over the lamina, medial, and inferior to the facet joint (Figure 1b,c). Successful PNS lead implant and stimulation of the medial branch nerves was evidenced by selective activation of the lumbar multifidi and documented in all participants by visualization of multifidi contraction under ultrasound, as well as reports of comfortable sensations covering the region of axial back pain. Following lead implantation and confirmation of successful medial branch nerve stimulation, the introducers were removed, leaving the percutaneous leads in the tissue, which were then secured with surgical glue and waterproof dressings. The percutaneous leads were connected to small wearable stimulators (SPRINT PNS System, SPR Therapeutics; Figure 1a) and a range of stimulation settings were customized for each participant to generate comfortable, cyclical activation of the multifidi, while allowing for individual adjustment of stimulation intensity levels within that range (stimulation parameters: frequency: 12 Hz; duty cycle: 50%; amplitude range: 0–30 mA; pulse duration range: 10–200 µs). Participants were instructed to use PNS for 6–12 h/day for up to 60 days, after which time, the leads were withdrawn during a clinic visit using gentle traction (i.e., without surgery). Participants were encouraged to continue most of their normal activities and asked to complete regular follow‐up visits during the 2‐month treatment period and long‐term up to 12 months after the PNS lead removal (see Figure 2, Subject Participation Flow Diagram). No participants received any additional interventions apart from percutaneous PNS for their LBP prior to the primary end point. Healthcare resource utilization was collected throughout the follow‐up period and will be analyzed in subsequent reports following completion of all long‐term follow‐up visits.

### Outcome measures

Participants recorded their daily average pain intensity (BPI‐5) levels and analgesic medication usage in weekly diaries at baseline and prior to each study visit. The primary end point was prospectively defined as the proportion of participants experiencing clinically significant reductions in chronic LBP, as evidenced by greater than or equal to 30% reduction in “average pain intensity” (BPI‐5) at the end of the 2‐month treatment (i.e., average across last week of stimulation) compared to baseline (i.e., average across week prior to stimulation). Sample size estimation was performed with a goal of generating a 95% confidence interval for the primary end point with a ±10%–11% margin of error, yielding a target sample size of ~ 90 enrolled before attrition of participants. Although enrollment was stopped at 89 participants (1 short of the target) in March 2020 due to nationwide restrictions and uncertainty related to the coronavirus disease 2019 (COVID‐19) pandemic, analysis of the completed dataset for the primary end point indicates that a ±10.6% margin of error (within the goal of 10%–11%) was achieved. Secondary, functional, and patient‐centric outcomes were assessed via validated survey instruments at baseline and follow‐up visits, including back pain‐related disability (Oswestry Disability Index [ODI]), pain interference (BPI, Question #9), patient global impression of change (PGIC), depression (BDI‐II), and health‐related quality of life (RAND‐36). The prospectively defined secondary end points included change in disability (proportion of participants experiencing ≥10‐point reduction in ODI), change in pain interference (proportion of participants experiencing ≥30% reduction in BPI‐9), PGIC, mean change in emotional state (depression, BDI‐II), and mean change in health‐related quality of life (RAND‐36). Primary and secondary end points were also analyzed at study intervals after end of treatment with PNS. Adverse events were collected throughout the duration of the clinical study.

At the time of manuscript submission, data collection, monitoring, and analysis of the primary end point and visits through 8 months after the start of treatment were complete, as all participants had completed the primary end point (2 months of PNS) and follow‐up visits through at least 6 months after PNS lead removal or been recorded as lost to follow‐up (Figure 2, Subject Participation Flow Diagram). Planned future publications will report the final results of the 14‐month follow‐up after all participants have completed the last follow‐up visit and data collection, monitoring, and analyses are complete.

### Statistical analysis and data handling

Statistical analyses were performed on data as observed from eligible participants who had completed visits through end of treatment with PNS (primary end point) and the follow‐up visit 8 months after start of treatment. Because prospective follow‐up remains ongoing for some participants, data thereafter (months 11–14 after start of treatment) are reported as observed and summarized, with statistical analyses to be reported in future publications pending completion of follow‐up. Data were analyzed using one‐way analysis of variance (ANOVA) with post hoc Tukey‐Kramer adjustment for multiple comparisons and *p* = 0.05 level of significance (SAS, Cary, North Carolina, USA). Data are shown as mean (SD), unless otherwise stated, and 95% confidence intervals (CIs) are reported as 95% CI (lower limit and upper limit).

## RESULTS

### Study participants

Participants provided informed consent and were assessed for eligibility to enroll in the study from June 2017 to March 2020. Of the 166 consenting participants, 67 were excluded during screening because they did not meet the prospectively defined eligibility criteria (Figure 2). Ten participants with a history of lumbar surgery were enrolled under this study protocol’s IRB approval as part of a prospectively designed substudy with revised exclusion criteria to allow prior surgery, planned to be analyzed, published, and presented separately from the primary group of participants, and are therefore not included in this report (Figure 2). Eighty‐nine participants were enrolled and underwent percutaneous PNS lead implantation for evaluation of the primary end point, and 8 enrolled participants were later found to be ineligible because they not meet the predefined eligibility criteria at baseline (e.g., due to unreported radicular leg pain, new medications at baseline, lumbar scoliosis, etc.), but were included in a sensitivity analysis to confirm there was no meaningful impact on the primary end point, and their data were included in the safety analysis. Of the 81 eligible patients who started PNS treatment, 7 participants were lost to follow‐up and 74 participants completed PNS treatment, providing data for evaluation of the primary end point. Of those 74 participants, 70 (95%) completed the 5‐month follow‐up visit (4 were lost to follow‐up) and 66 (89%) completed the 8‐month follow‐up visit. As described in the Methods—Statistical Analysis and Data Handling section, statistical tests were only conducted for visits where data collection was complete (i.e., *n* = 74 at the end of PNS treatment, primary end point; *n* = 70 at the 5‐month follow‐up; *n* = 66 at the 8‐month follow‐up), and data for subsequent timepoints are reported as observed, with 51 participants (i.e., 69% of those completing PNS treatment) having completed the 14‐month visit and others continuing through the prospective follow‐up period.

Table 1 shows the participants’ demographics and baseline information. At baseline, participants reported moderate to severe back pain, with an average back pain intensity of 6.1 (1.2; BPI‐5) and a worst back pain intensity of 7.6 (1.2; BPI‐3), moderate to severe back pain‐related disability, and substantial interference of pain on daily activities (Table 1). The most commonly documented back pain diagnoses and/or etiologies of pain included lumbar spondylosis (37%), degenerative disc disease (32%), and unknown/nonspecific chronic LBP (28%). Participants were required to have previously failed at least two types of LBP treatments, and the most commonly failed previous treatments for chronic back pain included non‐opioid analgesics (97% of participants), physical therapy (89%), opioid analgesics (67%), transcutaneous electrical nerves stimulation (TENS, 65%), chiropractic manipulation (61%), lumbar anesthetic or corticosteroid injections (57%), epidural injection (46%), and RFA (23%), suggesting that these prior treatments had only short‐term effects or were ineffective for these participants’ pain. Current opioid analgesic usage for back pain was reported by 27% of participants at baseline (*n* = 20/74), with a mean 32.0 mg morphine equivalent (MME) daily consumption among those reporting baseline opioid usage. The physician investigators were asked which therapies they would recommend for each participants’ axial back pain, had they not been participating in the clinical study, and which therapies could potentially be avoided or delayed by treatment using the PNS system. The most common treatments that would have been recommended for these participants were RFA (51%), spinal cord stimulation (26%), lumbar surgery (13%), and/or other/conventional treatment (26%; e.g., medication management, anesthetic injection, and other treatments). Percutaneous PNS leads were placed at the spinal level in the center of the region of pain for each participant, which was most commonly L4 (44% of leads) or L5 (41%), with 91% of participants receiving 2 leads placed bilaterally (i.e., one on each side, *n* = 67/74).

**TABLE 1:** Demographics and baseline information

Participant demographics (*n* = 74)	
Age (years)	56.3 (13.5)
21–44 years (% of population)	24%
45–64 years (% of population)	43%
≥65 years (% of population)	32%
BMI	29.4 (4.6)
LBP duration (years)	16.0 (13.0)
Sex (% female)	53%
Work status at baseline	
Currently working	45%
Retired (not due to health)	28%
Disabled due to back pain	8%
Unemployed	5%
Other (e.g., homemaker, on leave of absence, student, other)	12%
Baseline scores	
Baseline average pain (BPI‐5)	6.1 (1.2)
Baseline worst pain (BPI‐3)	7.6 (1.2)
Baseline disability (ODI)	38.5 (12.5)
Baseline pain interference (BPI‐9)	5.6 (2.1)
Baseline depression (BDI‐II)	8.5 (5.4)
Baseline opioid consumption (MME; among *n* = 20 taking opioids at baseline)	32.0 (37.1)

Note

Results shown as mean (SD) unless otherwise stated.

Abbreviations: BDI, Beck Depression Inventory; BMI, body mass index; BPI, Brief Pain Inventory; MME, mean morphine equivalent; ODI, Oswestry Disability Index; LBP, low back pain.

### Primary end point and reductions in pain intensity

**FIGURE 3:**
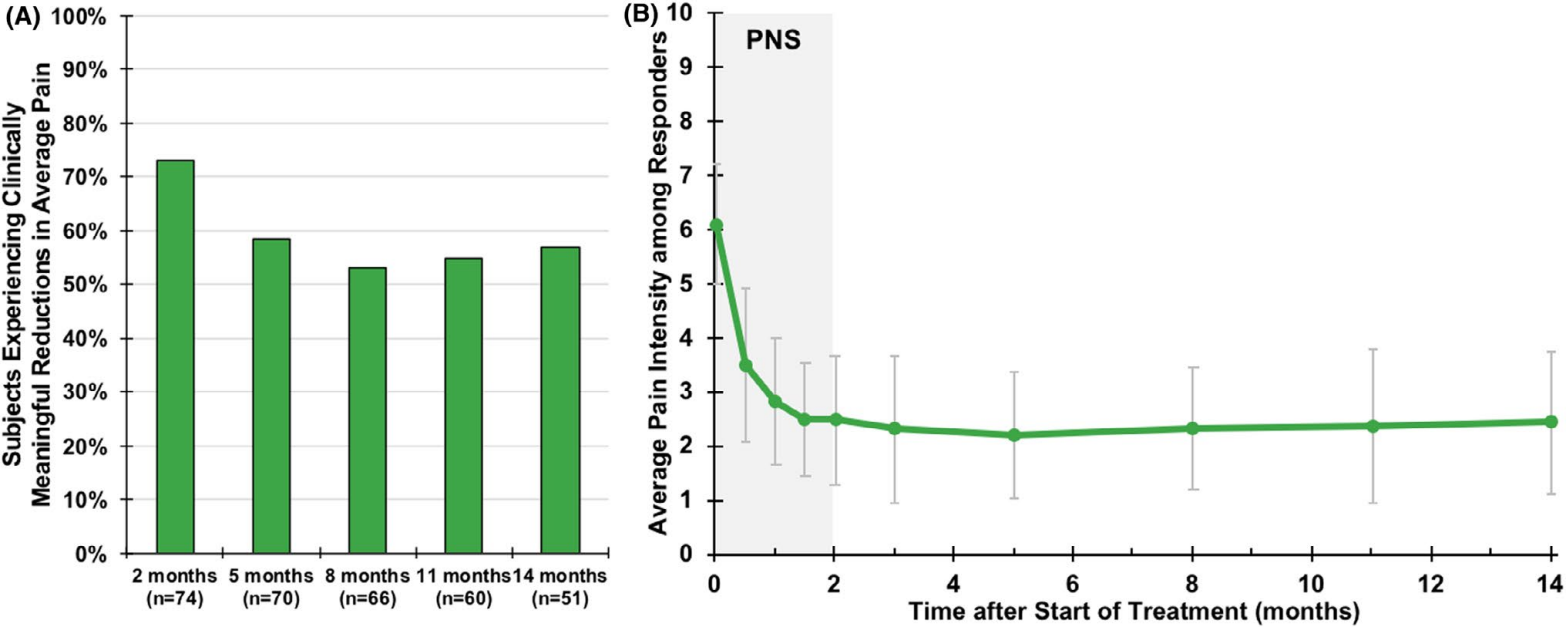
Reductions in average back pain intensity. (a) Shows the proportion of participants responding with clinically meaningful reductions in average pain intensity (Brief Pain Inventory, question 5 [BPI‐5]) over time. Data collection is complete for follow up visits through 8 months (including the primary end point at 2 months), with data reported thereafter (months 11–14) as observed, while prospective follow‐up is ongoing. (b) Shows the average pain intensity scores (mean ± SD) among responders. PNS, peripheral nerve stimulation

A majority of participants, 73% (*n* = 54/74, 95% CI [61.4%, 82.6%]) were responders for the primary end point, reporting clinically meaningful (≥30%) reductions in back pain intensity at the end of the 2‐month percutaneous PNS treatment (Figure 3), with an average 58% reduction in average back pain intensity (BPI‐5) among responders. Across all participants completing the primary end point, average back pain intensity (BPI‐5) was reduced with percutaneous PNS from baseline of 6.1 (1.2) to 3.3 (1.9) after 2 months (*n* = 74, *p* < 0.001; Table 2), and 43% (*n* = 32/74) experienced highly clinically meaningful (≥50%) reductions in pain intensity. A post hoc sensitivity analysis was performed to explore the effect of not including participants found not to meet the predefined eligibility criteria. When including those who did not meet the prospectively defined eligibility criteria (*n* = 8, Figure 2) in a post hoc analysis of the primary end point, 70% (*n* = 57/82, 95% CI [58.4%, 79.2%]) were responders for the primary end point, reporting clinically meaningful reductions in back pain intensity at the end of the 2‐month percutaneous PNS treatment, confirming that not including the ineligible participants did not have a meaningful impact the interpretation of the primary end point results. Additionally, these reductions in average back pain intensity remained significantly reduced to 3.6 (2.1) and 3.9 (2.1) long‐term after PNS lead removal (5 and 8 months after start of treatment, respectively, *p* < 0.001, Table 2). Whereas prospective follow‐up beyond 8 months is still ongoing, for those who had already completed the long‐term follow‐up visits (i.e., *n* = 51), the clinically meaningful reductions in average back pain intensity were sustained among a majority (57%) of participants through 14 months after the start of PNS (i.e., 12 months after PNS lead removal; Table 2, Figure 3).

**TABLE 2:** Statistically significant reductions in pain intensity, pain interference, and disability

Timepoint	Mean (SD)
Average pain intensity (BPI‐5)	
Baseline (*n* = 74)	6.1 (1.2)
2 months (*n* = 74)	3.3 (1.9)*
5 months (*n* = 70)	3.6 (2.1)*
8 months (*n* = 66)	3.9 (2.1)*
11 months (*n* = 60)	4.1 (2.4)^a^
14 months (*n* = 51)	3.9 (2.2)^a^
Oswestry Disability Index	
Baseline (*n* = 73)	38.5 (12.5)
2 months (*n* = 73)	22.9 (12.6)*
5 months (*n* = 70)	25.0 (14.7)*
8 months (*n* = 65)	27.5 (15.0)*
11 months (*n* = 58)	28.9 (15.7)^a^
14 months (*n* = 50)	29.5 (15.3)^a^
Pain interference (BPI‐9)	
Baseline (*n* = 74)	5.6 (2.1)
2 months (*n* = 73)	2.7 (2.0)*
5 months (*n* = 70)	3.0 (2.4)*
8 months (*n* = 65)	3.3 (2.4)*
11 months (*n* = 58)	3.7 (2.5)^a^
14 months (*n* = 50)	3.5 (2.3)^a^

Abbreviations: ANOVA, analysis of variance; BPI, Brief Pain Inventory.

^a^ Follow‐up is ongoing; data are reported as observed in months 11–14.

* *p* < 0.001, ANOVA with post hoc Tukey‐Kramer adjustment for multiple comparisons; statistical analyses were conducted only for timepoints where data collection was complete.

### Secondary end points and improvements in functional, patient‐centric outcomes

**FIGURE 4:**
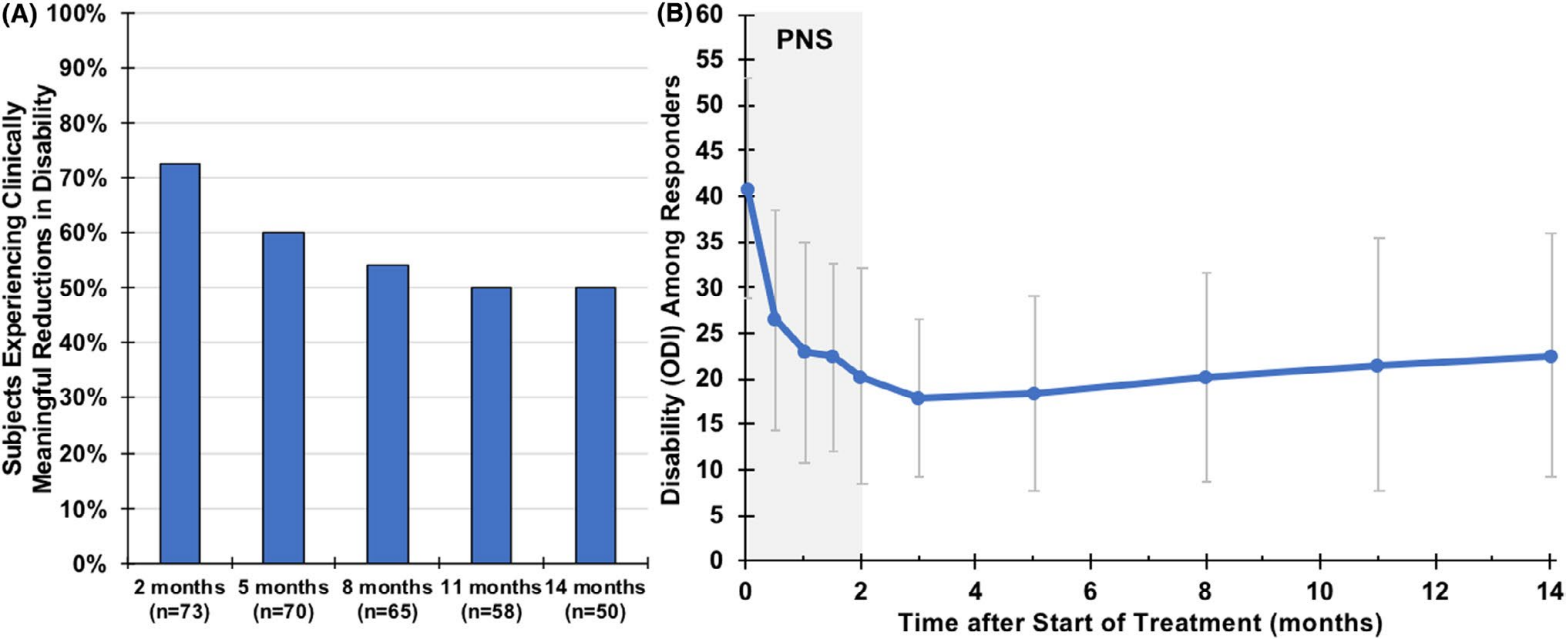
Reductions in back pain‐related disability. (a) Shows the proportion of participants responding with clinically meaningful reductions in back pain‐related disability (Oswestry Disability Index [ODI]) over time. Data collection is complete for visits through 8 months, with data reported thereafter (months 11–14) as observed, while prospective follow‐up is ongoing. (b) Shows the disability scores (mean ± SD) among responders. PNS, peripheral nerve stimulation

**FIGURE 5:**
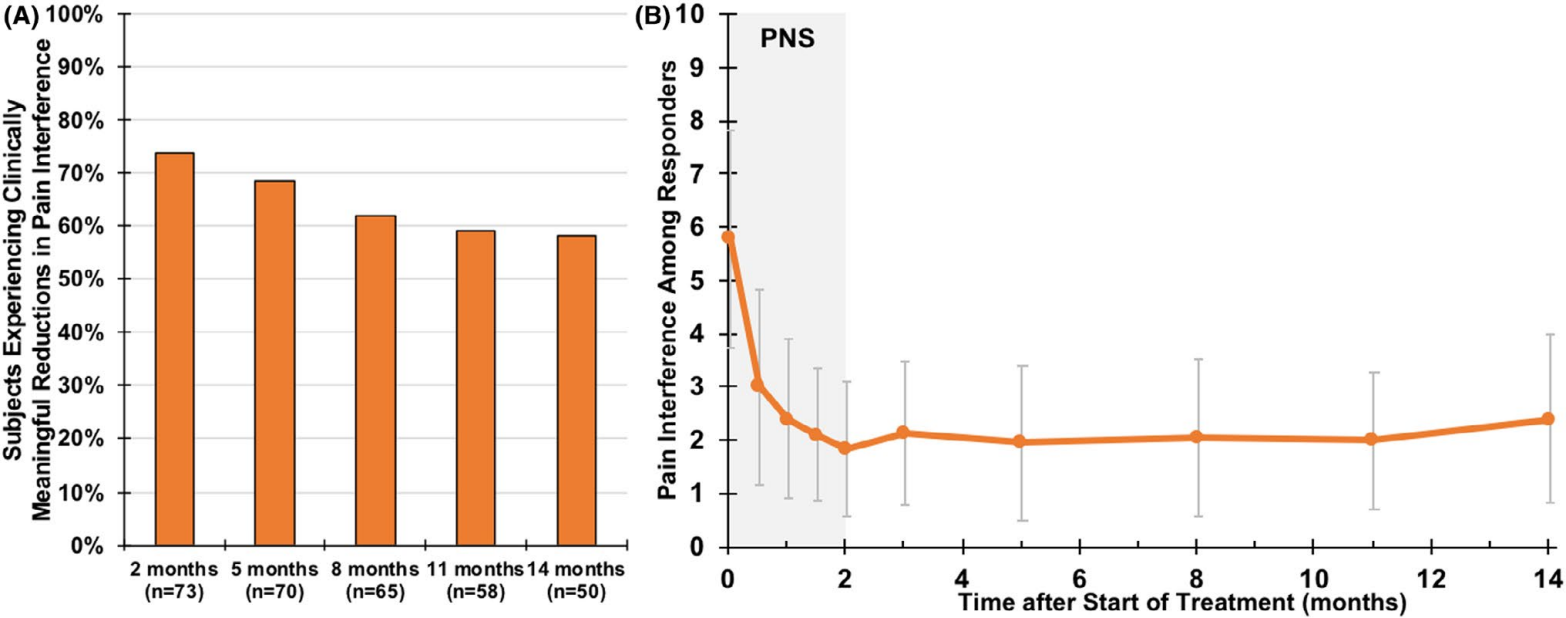
Reductions in pain interference. (a) Shows the proportion of participants responding with clinically meaningful reductions in pain interference (Brief Pain Inventory [BPI]‐9) over time. Data collection is complete for visits through 8 months, with data reported thereafter (months 11–14) as observed, while prospective follow up is ongoing. (b) Shows the pain interference scores (mean ± SD) among responders. PNS, peripheral nerve stimulation

Seventy‐three percent of participants (*n* = 53/73, 95% CI [60.9%, 82.4%]) experienced clinically meaningful reductions in back pain‐related disability (ODI, Figure 4, average 21‐point reduction among responders) at the end of the 2‐month percutaneous PNS treatment. Among participants providing data for this end point, the mean ODI score was reduced from 38.5 (12.5) at baseline to 23.3 (12.9) after 2 months of PNS, and 25.0 (14.7) and 27.5 (15.0) at the 5‐ and 8‐month follow‐up visits, respectively (*p* < 0.001; Table 2). A majority of participants, 73% (*n* = 53/73, 95% CI [60.9%, 82.4%]), also experienced clinically meaningful reductions in pain interference (BPI‐9, Figure 5, average 67% reduction among responders) at the end of the 2‐month percutaneous PNS treatment. The mean BPI‐9 score was reduced from 5.6 (2.1) at baseline to 2.7 (2.0) after 2 months of PNS, and 3.0 (2.4) and 3.3 (2.4) at the 5‐ and 8‐month follow up visits, respectively (*p* < 0.001; Table 2). Whereas follow‐up remains ongoing, for those who had already completed the long‐term follow‐up visits, the clinically meaningful reductions in disability and pain interference appeared to be sustained through 14 months after the start of PNS (i.e., 12 months after PNS lead removal; Table 2, Figures 4 and 5).

Of the 74 participants completing PNS treatment, 91% (*n* = 67/74, 95% CI [81.5%, 96.1%]) indicated their quality of life was improved at the end of the 2‐month percutaneous PNS treatment, with 59% (*n* = 44/74, 95% CI [47.4%, 70.7%]) reporting that they were at least “much improved” compared to baseline (PGIC a 7‐point scale of “very much worse” to “very much improved”). Overall, for participants completing the PNS treatment, the mean PGIC on their quality of life was “much improved.” Whereas follow‐up remains ongoing, among those who had already completed the long‐term follow‐up visits, preliminary results showed that 14 months after the start of PNS, a majority of participants continued to report quality of life improvements via PGIC attributed to PNS (51%, *n* = 26/51, 95% CI [36.6%, 65.3%]).

Among participants reporting opioid analgesic consumption at baseline (*n* = 20), a majority of participants reported reductions in opioid usage at the end of the 2‐month percutaneous PNS treatment (63%, *n* = 12/19; average 65% reduction among those reducing, equivalent to an average 15.1 MME reduction from 28.5 MME at baseline to 13.4 MME after PNS treatment among those reducing). At the 5‐month follow up visit, 65% reported reductions in opioid usage (*n* = 13/20; average 65% reduction among those reducing, equivalent to an average 15.6 MME reduction from 25.9 MME at baseline to 10.4 MME at 5 months among those reducing). At the 8‐month follow‐up visit, 61% reported reductions in opioid usage (*n* = 11/18, average 62% reduction among those reducing, equivalent to an average 18.6 MME reduction from 25.4 MME at baseline to 6.8 MME after PNS treatment among those reducing). Complete opioid cessation (100% reduction in opioid analgesic consumption) was reported by 21% (*n* = 4/19) after 2 months of PNS and 20% (*n* = 4/20) and 17% (*n* = 3/18) at the 5‐ and 8‐month follow‐up visits, respectively. Whereas follow‐up remains ongoing, for those taking opioids at baseline who had already completed the long‐term follow‐up visits, 57% (*n* = 8/14) reported reductions in daily opioid usage and 21% (*n* = 3/14) reported complete opioid cessation 14 months after the start of PNS.

All subscales of the health‐related quality of life survey RAND‐36 showed improvement with PNS compared to baseline (Table 3). The mean scores for RAND‐36 subscales of Pain, Physical Functioning, Role Limitations—Physical Health, Energy/Fatigue, Emotional Well‐being, and Social Functioning were each significantly increased (i.e., improved) between baseline and the end of the 2‐month PNS treatment (*n* = 73, *p* < 0.05; Table 3). The RAND‐36 subscales showing the largest improvements (i.e., increases in health score compared to baseline) with PNS compared to baseline were Role Limitations—Physical Health (128% improvement), Pain (62% improvement), and Physical Functioning (33% improvement). Beck Depression Inventory (BDI‐II) scores were significantly reduced from baseline to the end of the 2‐month percutaneous PNS treatment, as the mean depression score at baseline was 8.5 (5.4) and was reduced to 5.3 (5.2; 38% reduction) after 2 months of treatment (*n* = 73, *p* < 0.001).

**TABLE 3:** Results of RAND‐36 health‐related quality of life survey

	Physical functioning	Role limitations—physical health	Role limitations—emotional problems	Energy/fatigue	Emotional well‐being	Social functioning	Pain	General health
Baseline								
Mean	42.8	21.9	63.0	40.8	72.5	57.2	35.5	64.2
SD	22.6	35.6	43.2	20.6	17.2	26.2	17.2	21.8
End of treatment with PNS								
Mean	56.8**	50.0**	74.0	53.9**	79.7*	74.7**	57.6**	69.5
SD	24.0	40.4	39.8	19.8	15.0	22.6	20.6	19.8
Improvement with percutaneous PNS	14.0	28.1	11.0	13.2	7.2	17.5	22.1	5.2
	33%	128%	17%	32%	10%	31%	62%	8%

Abbreviation: PNS, peripheral nerve stimulation.

* *p* < 0.05

** *p* < 0.001.

### Adverse events

Table 4 summarizes the adverse events reported by participants throughout the duration of the study. All participants who were enrolled and underwent lead implantation are included in this safety analysis, including the 8 participants who not included in the primary end point analysis after they were found not to have not met the prospectively defined eligibility criteria at baseline and the 7 participants lost to follow‐up before the end of the 2 months PNS treatment period (Figure 2). There were no serious or unanticipated adverse events. Adverse events related to the device or procedure that did occur were all non‐serious (mild or moderate) and all adverse events were followed to resolution. The most common adverse events were mild skin irritation or pruritis (itching) at the site of the waterproof dressing or stimulator’s hydrogel mounting pad. One participant experienced a superficial skin infection at one lead exit site that was resolved by removal of the lead in the week prior to the end of treatment and use of an oral antibiotic.

**TABLE 4:** Adverse events among all participants receiving percutaneous PNS leads (*n* =89)

Category of adverse event	No. of adverse events	Adverse event severity		No. of participants affected
		Mild	Moderate	
Dermatological: skin irritation	34	27	7	28
Dermatological: pruritus	21	19	2	19
Dermatological: granuloma, discoloration, urticaria, other	8	5	3	7
Neurological: new pain	8	6	2	6
Neurological: worsening pain	5	5	0	5
Neurological: discomfort	5	3	2	5
Dermatological: infection^a^	2	0	2	2
Other: neurological/other	2	2	0	2
Other: cardiovascular (e.g., temporary vasovagal response)	1	1	0	1
Unable to determine relationship	5	5	0	5
Not study related (e.g., strep throat, urinary tract infection, other musculoskeletal, other pain, etc.)	22	3	19	12

Abbreviation: PNS, peripheral nerve stimulation.

^a^ One superficial infection at a lead exit site was resolved with removal of lead 1 week prior to end of treatment and an oral antibiotic (first reported in Deer et al.^44^). One skin infection at the location of the waterproof dressing was resolved with discontinuation of benzoin tincture (used to increase dressing adhesion and suspected to be cause of the irritation) and an oral antibiotic.

## DISCUSSION

This prospective multicenter case series study demonstrates the potential clinical utility of percutaneous PNS when applied to the medial branch nerves for the treatment of chronic axial LBP recalcitrant to nonsurgical treatments. This study evaluated percutaneous PNS in patients who had failed multiple prior treatments to determine if the results from earlier smaller single‐center studies were generalizable when performed by physicians with varied specialties and techniques, across a wide variety of centers (e.g., large academic centers, research institutions, and private practice clinics) and settings with rural and urban populations. Clinically meaningful and statistically significant reductions in pain, disability, and pain interference were reported by a majority of participants who completed the primary end point at 2 months and each of the subsequent follow‐up visits through 8 months after start of PNS. Whereas prospective follow‐up is still ongoing, among those who had completed the long‐term follow up visits, the reductions in pain and improvements in functional outcomes of disability and pain interference were sustained long‐term. Additional potential benefits of percutaneous PNS among participants with chronic axial back pain were observed as significant or substantial improvements in patient global impression of change, depression, and health‐related quality of life with PNS. Notably, the clinically meaningful improvements in pain and functional outcomes reported with temporary, 2‐month PNS treatment are consistent with the prior published clinical trials and case reports, suggesting percutaneous PNS offers an effective minimally invasive treatment alternative for patients with LBP who would have been recommended more destructive or invasive options, such as RFA, surgery, and permanently implanted neurostimulation or intrathecal drug delivery systems.

Percutaneous PNS, with its unique fine‐wire, open‐coil lead, minimally invasive lead implant procedure, and short‐term treatment period, was designed to be a safe, effective, non‐opioid, neurostimulation option for patients earlier in the treatment continuum. No serious or unanticipated adverse events were reported. The most common adverse events reported were mild skin irritation or pruritis (Table 4). One participant experienced a superficial skin infection after 7 weeks of stimulation at one lead exit site that was resolved after removal of the lead and use of an oral antibiotic (first reported in substudy of participants with prior radiofrequency ablation by Deer et al.^44^). Whereas the lead exit site infection was not confirmed by culture, a previous analysis of published neurostimulation safety data found that the coiled percutaneous leads have a statistically significantly lower risk of infection compared with noncoiled neurostimulation leads.^45^

### Proposed mechanism of action

Many chronic pain conditions, including LBP, are characterized by a persistent cycle of pain and functional disability that may be perpetuated by central sensitization and/or altered central pain processing, such as functional cortical reorganization.^46^, ^47^, ^48^, ^49^, ^50^, ^51^, ^52^, ^53^ Chronic axial LBP is often nonspecific (i.e., where an underlying etiology of pain is not easily identifiable and there is no clear source of nociceptive input or damage to the system) in a large majority of patients.^54^ These patients commonly report abnormal central pain processing, as evidenced by hypersensitivity to normal stimuli or the perception of pain long after an injury has healed,^48^, ^55^, ^56^, ^57^, ^58^ and effective treatments have previously been shown to be correlated with the reversal of these central pain processing abnormalities.^49^, ^57^, ^59^ The proposed analgesic mechanism of action responsible for sustained relief of chronic LBP following a temporary percutaneous PNS treatment is modulation of the underlying central sensitization through peripherally induced reconditioning of the central nervous system.^60^, ^61^ Percutaneous PNS is believed to produce robust neural signals in sensory (afferent) fibers focal to the region of back pain that engage the gate mechanism and decrease central pain signals, both directly and indirectly through stimulation of efferent fibers that activate muscles generating a reflex arc of proprioceptive afferent signals.^34^, ^62^, ^63^ These focal and robust signals are thought to help normalize or reverse membrane hyperexcitability of circuits in nociceptive and neuropathic pathways, disrupting the cycle of centrally maintained pain and permitting greater levels of physical activity in conjunction with prolonged decreases in pain signals, pain processing, and pain sensation. With increased levels of physical activity and reduced pain, activity dependent neuroplasticity is thought to maintain reductions in pain well after the active stimulation period has ended, as evidenced by the sustained pain reduction after the end of the 60‐day treatment. Altogether, percutaneous PNS applied to the medial branch nerves may generate robust neural signals that are focal to the area of lumbar pain and capable of modulating the maladaptive central pain processing states underlying nonspecific axial LBP, possibly explaining how a targeted, temporary treatment could be used to treat a broad population of chronic axial back pain.

### Limitations

Key limitations of this study include that it was not a randomized trial, did not include a control group, and is not yet complete (i.e., prospective follow‐up beyond 8 months remains ongoing). The prospective, multicenter case series study was designed to address selection bias by enrolling participants from a variety of clinical care settings across rural and urban populations, including large academic centers, research institutions, and private practice clinics, with varied physician investigator specialties, such as anesthesiology, pain medicine, physical medicine and rehabilitation, and neurosurgery. Because the objective of this study was to evaluate percutaneous PNS in patients who had failed multiple prior treatments to determine if the results from earlier smaller single‐center studies were generalizable when performed by physicians with varied specialties and techniques across a wide variety of centers, the study was designed as a case series with a single arm (although a possible limitation of this study was that outliers in responses to previous treatments could have impacted the results). Although the findings from this study would support a future randomized controlled study in patients with LBP to further address some of these limitations, 3 prior randomized controlled trials have demonstrated that temporary percutaneous PNS can produce clinically meaningful and statistically significant sustained effects relative to control groups in other chronic pain populations.^24^, ^27^, ^33^, ^64^

The results of the present multicenter clinical study and previous clinical trials, coupled with the consistent safety profile of percutaneous PNS, suggest that patients with chronic axial LBP who have failed multiple prior treatments may receive significant benefit from percutaneous medial branch PNS without the invasiveness and accompanying complications, costs, and risks of more invasive or destructive therapies and/or permanently implanted systems.

## CONCLUSIONS

This work explored the use of percutaneous PNS in a prospective multicenter case series study among participants from rural and urban populations with chronic axial LBP who had varied proposed etiologies of pain, had failed multiple prior treatments, and underwent treatment by physicians with varied specialties and techniques at a wide variety of centers (e.g., large academic centers, research institutions, and private practice clinics). Seventy‐three percent of participants were successes for the prospectively defined primary end point, reporting clinically significant reductions in back pain intensity at the end of the 2‐month percutaneous PNS treatment (*n* = 54/74), and a majority reported sustained relief through timepoints for which data collection is complete (8 months after start of treatment). Participants also reported clinically meaningful and statistically significant improvements in functional outcomes, as measured by disability and pain interference; commensurate improvements in other outcomes, such as depression, opioid analgesic consumption, and health‐related quality of life were also noted with percutaneous PNS. Whereas prospective follow‐up beyond 8 months is still ongoing, among a majority of those who had already completed the long‐term follow‐up visits, the clinically meaningful benefits experienced by participants were sustained long‐term, suggesting that a percutaneous 60‐day medial branch PNS treatment has the potential to serve as an effective, nondestructive neuromodulation option for patients with recalcitrant chronic axial back pain.

## CONFLICT OF INTEREST

C.A.G., M.J.D., T.J.H., S.L., M.J.D.p., T.R.D., W.G., A.H.B., P.K.S., and K.A. are physician investigators with clinical research sponsored by SPR Therapeutics. C.A.G., M.J.D., S.L., T.R.D., and S.P.C. are consultants for SPR, and M.J.D. and T.R.D. have equity ownership in SPR Therapeutics. M.J.M.g. and J.W.B. are employees of SPR Therapeutics with equity ownership.

## ENDNOTE

^1^
Whereas the primary study cohort excluded patients with prior lumbar surgery, patients with a history of lumbar surgery were enrolled under this IRB approval in a prospectively designed substudy with revised eligibility criteria, designed to be analyzed separately, and reported independently from the primary end point analysis of participants without a history of lumbar surgery (for more information, see “Results—Study Participants” and Figure 2).
